# Enhancing Li-S Battery Kinetics via Cation-Engineered Al^3+^/Fe^3+^-Substituted Co_3_O_4_ Spinels

**DOI:** 10.3390/ma19020326

**Published:** 2026-01-13

**Authors:** Zhiying Lin, Mingyu Wang, Wen Fu, Zhixin Gu, Zhenkai Yang, Kai Guan, Zaixing Yang, Lulu Wang, Wenjun Wang, Kaixing Zhu

**Affiliations:** 1 College of Electromechanical Engineering, Qingdao University of Science and Technology, Qingdao 266061, China; 2 School of Physics, Shandong University, Jinan 250100, China; 3 Institute of Physics, Chinese Academy of Sciences, Beijing 100190, China

**Keywords:** cation substitution, spinel oxides, lithium polysulfides, lithium-sulfur batteries

## Abstract

Lithium–sulfur (Li-S) batteries promise high energy density and low cost but are hindered by polysulfide shuttling, sluggish redox kinetics, poor sulfur conductivity, and lithium dendrite formation. Here, a targeted cation-substitution strategy is applied to Co_3_O_4_ spinels by replacing octahedral Co^3+^ sites with trivalent Al^3+^ or Fe^3+^, generating Al_2_CoO_4_ and Fe_2_CoO_4_ with exclusively tetrahedral Co^2+^ sites. Structural characterizations confirm the reconstructed cationic environments, and electrochemical analyses show that both substituted spinels surpass pristine Co_3_O_4_ in LiPS adsorption and catalytic activity, with Al_2_CoO_4_ delivering the strongest LiPS binding, fastest Li^+^ transport, and most efficient redox conversion. As a result, Li-S cells equipped with Al_2_CoO_4_-modified separators exhibit an initial capacity of 1327.5 mAh g^−1^ at 0.1C, maintain 883.3 mAh g^−1^ after 200 cycles, and deliver 958.6 mAh g^−1^ at 1C with an ultralow decay rate of 0.034% per cycle over 1000 cycles. These findings demonstrate that selective Co-site substitution effectively tailors spinel chemistry to boost polysulfide conversion kinetics, ion transport, and long-term cycling stability in high-performance Li-S batteries.

## 1. Introduction

The rapid growth of portable electronics, electric vehicles, and grid-scale energy storage has intensified the demand for next-generation rechargeable batteries featuring higher energy density and lower cost [1,2]. Among the many candidates, lithium–sulfur (Li-S) batteries have attracted extensive attention due to their ultrahigh theoretical energy density (2600 Wh kg^−1^), natural abundance of sulfur, and environmental friendliness [3,4,5,6]. Despite these advantages, their practical deployment is severely constrained by the intrinsic insulating nature of sulfur and Li_2_S, uncontrolled lithium dendrite formation, and the dissolution–migration of lithium polysulfides (LiPSs) that induces the notorious shuttle effect [7]. These challenges become even more critical at high sulfur loading and low electrolyte/sulfur (E/S) ratios, where elevated concentrations of soluble LiPSs trigger rapid capacity fading and poor sulfur utilization [8,9,10].

Functional separator engineering has emerged as an effective approach to overcome these limitations by regulating LiPS transport and promoting their redox conversion. The separator must maintain electronic insulation while allowing efficient Li^+^ migration, and incorporating functional interlayers—such as carbon materials, metal oxides, or conductive polymers—has proven effective in trapping LiPSs and accelerating their chemical transformation [11,12,13,14,15]. Catalytically active interlayers are particularly advantageous, as they facilitate the rapid conversion of long-chain LiPSs into insoluble Li_2_S_2_/Li_2_S, thereby improving redox kinetics, reactivating electrically isolated sulfur species, and supporting high areal capacities under practical operating conditions [16,17].

Among various catalytic candidates, metal oxides stand out due to their strong polar interactions with LiPSs, abundant Lewis acidic sites, and defect-rich surfaces. Their high mechanical and packing density also benefits the development of high-volumetric-energy Li-S systems. Bimetallic oxides, in particular, offer cooperative adsorption–catalysis effects and multiple accessible active sites, enabling excellent rate capability and long-term cycling. Representative examples, including NiCo_2_O_4_, MnO-based materials, and MnO_2_ composites, have demonstrated substantial improvements in LiPS conversion kinetics and the suppression of shuttle behavior [18,19,20,21,22]. Spinel Co_3_O_4_ is especially promising owing to its well-defined cation arrangement, environmental compatibility, and intrinsic catalytic activity [23]. Its AB_2_O_4_ structure consists of tetrahedral Co^2+^ and octahedral Co^3+^ sites, whose distinct coordination environments critically influence its adsorption characteristics and catalytic pathways in electrochemical reactions [24,25,26,27,28,29,30]. Importantly, the catalytic properties of Co_3_O_4_ can be deliberately tuned through selective substitution of foreign metal cations, which modulates its electronic structure, defect configuration, and interaction strength with reaction intermediates [31,32,33,34]. This site-specific tuning offers a powerful strategy for designing high-efficiency catalytic hosts tailored for Li-S chemistry.

In this work, we realize the selective substitution of trivalent metal cations (Al^3+^, Fe^3+^) into the octahedral Co^3+^ sites of spinel Co_3_O_4_ through a sol–gel route, forming Al_2_CoO_4_ and Fe_2_CoO_4_ with reconfigured crystal frameworks. Using Al_2_CoO_4_ as a functional separator modification layer, we systematically evaluate its LiPS anchoring capability, catalytic conversion efficiency, Li^+^ transport behavior, and electrochemical performance. The findings demonstrate that site-tailored spinel oxides can serve as high-performance multifunctional separator coatings, providing new insights into catalyst design for high-energy-density Li-S batteries.

## 2. Experimental

### 2.1. Synthesis of Co_3_O_4_, Al_2_CoO_4_, and Fe_2_CoO_4_

Co_3_O_4_ and its Al/Fe-substituted derivatives were synthesized via a sol–gel method. Typically, 16.5 mmol Co(NO_3_)_2_·6H_2_O, 33.75 mmol ammonium citrate tribasic (ACT), and 24 mL ethylene glycol (EG) were dissolved in 25 mL deionized water (DI) under stirring to form a clear solution. Citric acid was subsequently introduced as a chelating agent to stabilize the metal–ligand complex and promote homogeneous gel formation. The resulting sol was heated to 120 °C to induce dehydration and gelation. After drying, the obtained precursor was calcined in air at 800 °C for 2 h at a ramp rate of 5 °C min^−1^, yielding Co_3_O_4_.

For Al_2_CoO_4_, Co(NO_3_)_2_·6H_2_O was partially replaced by Al(NO_3_)_3_·9H_2_O at a molar ratio of 1:2, while Fe_2_CoO_4_ was obtained by substituting Co(NO_3_)_2_·6H_2_O with FeCl_3_ at the same ratio. All final oxides were thoroughly ground and stored for subsequent use.

### 2.2. Fabrication of Co_3_O_4_-, Al_2_CoO_4_-, and Fe_2_CoO_4_-Modified Separators

For separator modification, the respective oxide powders were mixed with conductive carbon black and polyvinylidene fluoride (PVDF) at a mass ratio of 7:2:1. The mixture was manually ground for 30 min, followed by the addition of N-methyl-2-pyrrolidone (NMP) to form a homogeneous slurry. The slurry was coated uniformly onto commercial polypropylene (PP) separators using a doctor blade and dried under vacuum at 65 °C overnight. The resulting films were punched into 19 mm discs to prepare the modified separators.

### 2.3. Preparation of S/CNT Cathode

The sulfur/carbon nanotube (S/CNT) composite was prepared via a melt-diffusion method. CNTs and sulfur were mixed at a mass ratio of 1:2, followed by the addition of carbon disulfide (CS_2_). The mixture was ultrasonicated at 70 °C until viscous and subsequently dried at 70 °C for 12 h. The dried powder was then heated under argon at 155 °C for 10 h to infuse sulfur, followed by an additional treatment at 200 °C for 2 h. After natural cooling, the S/CNT composite was obtained.

For cathode fabrication, the S/CNT composite was blended with carbon black and PVDF at a mass ratio of 7:2:1 and ground for 30 min. NMP was added to form a uniform slurry, which was cast onto aluminum foil and dried under vacuum at 65 °C overnight. The electrodes were punched into 16 mm discs for cell assembly.

### 2.4. Assembly of Cells

Coin cells (CR2032-type) were assembled for electrochemical evaluation within an Ar-filled glovebox. Prior to assembly, the cathode was prepared by depositing 15 µL of 0.2 M Li_2_S_6_ solution onto its surface, followed by complete solvent evaporation. The cell configuration consisted of the Li_2_S_6_-loaded cathode, a lithium metal foil counter electrode, a Celgard 2400 polypropylene separator (Charlotte, NC, USA), and an electrolyte comprising 1 M LiTFSI in a 1:1 (*v*/*v*) mixture of 1,3-Dioxolane (DOL) and 1,2-Dimethoxyethane (DME) solution with 2 wt%LiNO_3_ additive. The electrolyte amount was controlled to maintain an electrolyte-to-sulfur (E/S) ratio of 30 µL mg^−1^.

### 2.5. Lithium Polysulfide Adsorption Test

A 5 mM Li_2_S_6_ solution was prepared by dissolving Li_2_S and sulfur in a molar ratio of 1:5 in DOL/DME (1:1 *v*/*v*) under argon for 24 h. Subsequently, 4 mL of this solution was added to vials containing 20 mg of Co_3_O_4_, Al_2_CoO_4_, or Fe_2_CoO_4_. After standing for 12 h, digital photographs were taken, and the supernatants were collected for UV-Vis spectroscopy to evaluate polysulfide adsorption capacity.

### 2.6. Polysulfide Diffusion Test

An H-type glass cell was used to examine the polysulfide diffusion-blocking capability of the modified separators. The separator was clamped between the two chambers, with one chamber filled with 5 mM Li_2_S_6_ solution and the other with pure DOL/DME solvent (1:1 *v*/*v*). The color changes in both chambers were photographed after 10 min, 12 h, and 24 h.

### 2.7. Lithium Sulfide (Li_2_S) Deposition Test

Li_2_S nucleation behavior was assessed in coin cells assembled with an S/CNT working electrode, lithium metal counter electrode, and the modified separator. A total of 40 μL electrolyte was added: 20 μL blank electrolyte on the anode side and 20 μL of 0.5 M Li_2_S_8_-containing electrolyte on the cathode side. The cell was discharged galvanostatically at 0.112 mA to 2.15 V to ensure a consistent initial state dominated by short-chain polysulfides (e.g., Li_2_S_4_), followed by a potentiostatic discharge at 2.05 V for 50,000 s to specifically investigate the kinetics of Li_2_S nucleation and growth under a controlled potential.

### 2.8. Electrochemical Testing

Galvanostatic charge–discharge tests (GCD) are carried out in the potential range of 1.7–2.8 V to evaluate the specific capacity and cycling stability of batteries on a LAND CT2001A system. Cyclic voltammetry (CV) measurements are performed over the potential range of 1.7 to 2.8 V at scan rates of 0.1–0.5 mV s^−1^ to investigate the reversibility of the electrode reaction. Electrochemical impedance spectroscopy tests (EIS) are conducted in the frequency range of 0.01 Hz to 10^5^ kHz with an AC amplitude of 5 mV to characterize the charge transfer kinetics.

### 2.9. Material Characterization

The crystal structures of the materials were analyzed by X-ray diffraction (XRD) on a Rigaku Ultima IV diffractometer with Cu K radiation (λ = 1.5406 Å), operating at 40 kV and 40 mA. Data were collected in the 2θ range of 20–80° with a scanning rate of 10°/min. The sample morphologies were examined using scanning electron microscopy (SEM, Hitachi Regulus 8100, Hitachi High-Tech Corporation, Tokyo, Japan) and transmission electron microscopy (TEM, FEI Talos F200x, Thermo Fisher Scientific, Brno, Czech Republic). Surface chemical states were investigated by X-ray photoelectron spectroscopy (XPS) on a Thermo Scientific K-Alpha spectrometer (Thermo Fisher Scientific, East Grinstead, UK) with a monochromatic Al Kα X-ray source. The adsorption of Li_2_S_6_ was evaluated by monitoring the UV-Vis absorption spectra of the DOL/DME solutions using a Persee TU-1810 spectrophotometer (Persee, Beijing, China).

## 3. Results and Discussion

### 3.1. Phase Analysis

**Figure 1:**
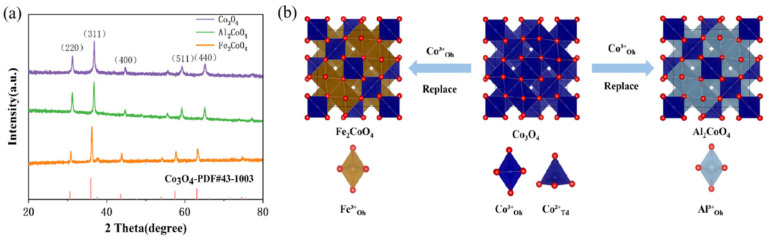
(**a**) XRD patterns of Co_3_O_4_, Al_2_CoO_4_ and Fe_2_CoO_4_. (**b**) Schematic diagram of the synthesis principle of Al_2_CoO_4_ and Fe_2_CoO_4_.

XRD was employed to investigate the influence of Al^3+^ and Fe^3+^ substitution at Co^3+^ sites on the crystalline phase of Co_3_O_4_. As shown in Figure 1a, all diffraction peaks of Co_3_O_4_, Al_2_CoO_4_, and Fe_2_CoO_4_ can be unambiguously indexed to the standard spinel phase (PDF# 43-1003), indicating that the introduction of Al^3+^ or Fe^3+^ does not alter the parent cubic spinel framework. The standard structures for Co_3_O_4_, Al_2_CoO_4_, and Fe_2_CoO_4_ are depicted in Figure 1b. The XRD patterns show that the diffraction peaks of Al_2_CoO_4_ almost overlap with those of Co_3_O_4_, suggesting negligible lattice contraction due to the similar ionic radii of Al^3+^ (0.535 Å) and Co^3+^ (0.545 Å). In contrast, the peaks of Fe_2_CoO_4_ exhibit a systematic shift towards lower angles, indicative of a slight lattice expansion originating from the larger ionic radius of Fe^3+^ (0.645 Å). Nevertheless, this change is uniform, and all diffraction peaks can be unequivocally indexed to a single spinel phase, demonstrating that the substitution is isovalent and does not compromise the overall integrity of the crystal structure.

**Figure 2:**
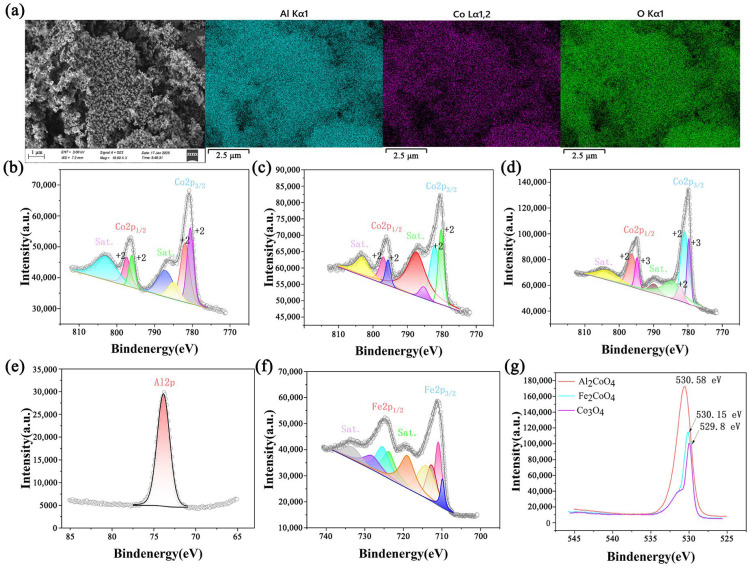
(**a**) SEM image of Al_2_CoO_4_ and Elemental mapping images of Al, Co and O. Co 2p XPS fine spectra of Al_2_CoO_4_ (**b**), Fe_2_CoO_4_ (**c**) and Co_3_O_4_ (**d**). (**e**) Al 2p XPS fine spectrum of Al_2_CoO_4_. (**f**) Fe 2p XPS fine spectrum of Fe_2_CoO_4_. (**g**) Comparison plots of XPS fine spectra of different materials, O 1s.

To further elucidate the cation distributions and chemical states, EDS-mapping and XPS analyses were conducted on Al_2_CoO_4_ powder. As illustrated in Figure 2a, the Al_2_CoO_4_ nanoparticles possess an irregular shape. Furthermore, Al, Co, and O elements are uniformly distributed in Al_2_CoO_4_. The Shirley method is used to eliminate interference from inelastic scattering effects. For peak modeling, a Lorentzian–Gaussian (L/G) mixed function with an L/G ratio of 0.3 is adopted for all deconvoluted peaks, a widely accepted standard for Co 2p spectral analysis. The binding energy difference between Co 2p_3_/_2_ and Co 2p_1_/_2_ is fixed at 15.0 eV (a canonical constraint for cobalt oxides), and the full width at half maximum (FWHM) values of peaks corresponding to the same valence state are constrained to be consistent to guarantee physical meaningfulness. Quantitative analysis results revealed that for Co_3_O_4_, Co^3+^ and Co^2+^ accounted for 65% and 35% of the total Co species, respectively, which is consistent with its mixed-valence spinel structure. In contrast, only Co^2+^-related peaks (including main peaks and satellite peaks) were detected in Al_2_CoO_4_ and Fe_2_CoO_4_, with no observable Co^3+^ signals (detection limit < 0.5%). This confirms that the octahedral Co^3+^ sites in the parent Co_3_O_4_ were fully substituted by Al^3+^ or Fe^3+^, resulting in the exclusive formation of tetrahedral Co^2+^ configurations in the substituted spinels. The XPS spectra of Co_3_O_4_ exhibit the typical Co 2p_3_/_2_ and Co 2p_1_/_2_ doublets, accompanied by satellite features at 782.2, 785.2, and 789.5 eV. The coexistence of Co^2+^ and Co^3+^ is consistent with the canonical mixed-valence nature of spinel Co_3_O_4_. In contrast, the Co 2p spectra of Al_2_CoO_4_ and Fe_2_CoO_4_ show deconvoluted peaks centered at ~780.0, 782.1, 785.5, and 786.5 eV, which align well with the characteristic binding energies of Co^2+^. No Co^3+^-related features are detected, demonstrating that Al^3+^ or Fe^3+^ substitution fully suppresses the formation of octahedral Co^3+^, resulting in spinels containing exclusively tetrahedral Co^2+^ sites. The Al 2p and Fe 2p spectra (Figure 2e,f) further verify the +3 oxidation states of the substituted Al and Fe ions, confirming their stable incorporation into the spinel lattice. In the Al 2p spectrum of Al_2_CoO_4_ (Figure 2e), a single peak is observed at a binding energy of 73.88 eV, characteristic of Al^3+^ in oxide environments [35]. Similarly, the Fe 2p spectrum of Fe_2_CoO_4_ (Figure 2f) shows primary peaks for Fe 2p_3_/_2_ and Fe 2p_1_/_2_ at 710.55 eV and 725.2 eV, respectively, accompanied by distinct satellite peaks. This signature is unequivocally assigned to high-spin Fe^3+^ [36]. The absence of peaks corresponding to lower oxidation states (e.g., Fe^2+^ or metallic Fe) confirms the phase-pure incorporation of trivalent cations into the spinel structure. Oxygen chemical-state analysis provides additional insight (Figure 2g), and the O 1s fitting was also performed using the Shirley background subtraction and the Lorentz–Gauss (L/G = 0.3) hybrid function. All spectra show a single symmetrical peak, indicating that only lattice oxygen (O^2−^) is present in the spinel, with no adsorbed oxygen or hydroxyl groups. The O 1s peak of Co_3_O_4_ appears at 529.8 eV, while those of Al_2_CoO_4_ and Fe_2_CoO_4_ shift to higher binding energies. This shift is attributed to the stronger Co^2+^-O interaction compared to Co^3+^-O bonding, which reflects the absence of octahedral Co^3+^ in the substituted spinels [37]. The combined XRD and XPS results confirm the successful synthesis of three cobalt-based spinels with distinctly different cationic configurations. Co_3_O_4_ contains both Co^2+^ at tetrahedral sites and Co^3+^ at octahedral sites, consistent with its mixed-valence spinel structure. In contrast, Al_2_CoO_4_ and Fe_2_CoO_4_ exhibit only tetrahedral Co^2+^, as the introduced Al^3+^ or Fe^3+^ fully replaces the octahedral Co^3+^. This controllable cation redistribution provides a solid foundation for tuning the electronic structure and interfacial chemistry of the materials.

### 3.2. Electrochemical Performance Analysis

**Figure 3:**
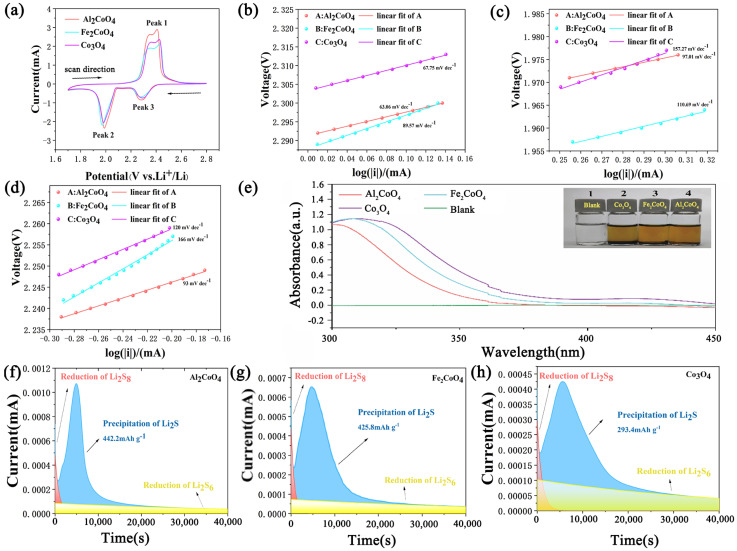
(**a**) CV Curve at 0.1 mV s^−1^. (**b**–**d**) Tafel plots of peak 1 (**c**), peak 2 (**d**) and peak 3 (**b**). (**e**) UV-Vis absorption spectra (inset: the optical image of visualized adsorption of Li_2_S_6_ by Al_2_CoO_4_, Fe_2_CoO_4_ and Co_3_O_4_). Fitted current-time transients during potentiostatic Li_2_S deposition at 2.05 V for (**f**) Al_2_CoO_4_, (**g**) Fe_2_CoO_4_, and (**h**) Co_3_O_4_ modified cells.

To elucidate the catalytic benefits attributed to tetrahedral Co^2+^ sites, a comprehensive electrochemical assessment of the three spinel oxides was conducted, as summarized in Figure 3. The CV profiles recorded at 0.1 mV s^−1^ (Figure 3a) exhibit one oxidation peak and two reduction peaks for all samples, corresponding to the stepwise transformation of S_8_ to Li_2_S and its reversible oxidation. The cyclic voltammetry profile reveals characteristic redox processes associated with the sulfur cathode. The oxidation peak at the higher potential (Peak 1) corresponds to the conversion of soluble long-chain polysulfides (Li_2_S_4_/Li_2_S_6_) to elemental sulfur (S_8_). During discharge, the primary reduction peak (Peak 3) signifies the reverse reaction, i.e., the reduction of S_8_ back to soluble long-chain polysulfides (Li_2_S_n_, 4 ≤ n ≤ 8). Subsequently, the reduction peak at the lower potential (Peak 2) is attributed to the solid–liquid reduction, where these soluble polysulfides are further reduced to insoluble solid discharge products, namely Li_2_S_2_ and Li_2_S. The negative shift in Peak 1 for Al_2_CoO_4_ indicates that the oxidation of polysulfides (Li_2_S_n_→S_8_) occurs at a lower overpotential. This is a classic signature of enhanced catalytic activity, meaning the catalyst significantly lowers the energy barrier for this oxidation step, facilitating the reverse reaction during charging. The positive shift and the remarkable increase in the current density of Peak 2 for Al_2_CoO_4_ are critically important. The shift suggests a reduced nucleation barrier for the deposition of Li_2_S. The dramatically higher current intensity signifies much faster kinetics for the conversion of soluble LiPS to insoluble Li_2_S_2_/Li_2_S. This is the core process for effectively trapping polysulfides and suppressing the shuttle effect. The enhanced intensity directly correlates with the higher Li_2_S deposition capacity measured in our potentiostatic discharge experiments (Figure 3f). The fact that Peak 3 remains relatively unchanged in position across all three materials suggests that the initial reduction of S_8_ to long-chain LiPS is less sensitive to the specific cation substitution in the spinel structure. The kinetics of this initial dissolution step may be inherently fast or governed by different factors. Notably, Al_2_CoO_4_ delivers the highest redox current responses and the smallest potential polarization, indicating accelerated sulfur redox kinetics and improved reaction reversibility. This enhancement suggests that Al_2_CoO_4_ effectively promotes the interfacial conversion of lithium polysulfides and facilitates higher sulfur utilization.

To further quantify the kinetic differences, Tafel polarization analysis was conducted (Figure 3b–d). Al_2_CoO_4_ consistently exhibits the lowest Tafel slopes among the three materials. For the oxidation process (Peak 1), Al_2_CoO_4_ exhibits a lower Tafel slope (63.06 mV dec^−1^) compared to that of Co_3_O_4_ (67.75 mV dec^−1^), indicating a reduced activation energy barrier for the oxidation of Li_2_S to S_8_ and thus accelerated charge transfer kinetics. For the reduction processes, the Tafel slopes corresponding to Peaks 2 and 3 for Al_2_CoO_4_ (97.01 and 93 mV dec^−1^, respectively) are substantially lower than those for Co_3_O_4_ (157.27 and 120 mV dec^−1^). This marked decrease confirms that Al_2_CoO_4_ can more effectively catalyze the conversion of lithium polysulfides, substantially enhancing the overall electrocatalytic performance of the cathode, and scalable fabrication of Ni(OH)_2_/carbon/polypropylene separators for high-performance Li-S batteries [38]. These results correlate well with the CV behavior and collectively demonstrate the strongest electrocatalytic activity of Al_2_CoO_4_ toward polysulfide conversion.

The polysulfide affinity of the three materials was further assessed through UV-Vis adsorption tests and separator diffusion experiments. As illustrated in Figure 3e, the Li_2_S_6_ solution treated with Al_2_CoO_4_ exhibits the most pronounced decrease in absorbance intensity, indicating the strongest adsorption capability. Visual observations further confirm this trend: the Li_2_S_6_ solution in contact with Al_2_CoO_4_ becomes significantly lighter in color than those exposed to Co_3_O_4_ or Fe_2_CoO_4_. Such strong adsorption helps suppress polysulfide migration and contributes to stable interfacial chemistry.

Since the conversion from Li_2_S_4_ to Li_2_S involves a challenging liquid-to-solid phase transition, and this step contributes approximately 75% of the total discharge capacity in Li-S batteries, investigating the nucleation and deposition mechanisms of Li_2_S is crucial for optimizing battery performance and enhancing practical capacity. Therefore, we systematically studied the nucleation and growth processes of solid Li_2_S on different electrodes using potentiostatic deposition. The assembled cells were first pre-discharged at a low current of 0.112 mA to 2.15 V, a step that converts long-chain polysulfides to Li_2_S_4_. Subsequently, the cells were held at 2.05 V for potentiostatic discharge, during which Li_2_S_4_ is reduced to Li_2_S. The discharge process was considered complete when the current became negligible (below 0.01 mA), indicating that all active material had been converted to Li_2_S (Figure 3f–h). Based on Faraday’s law, the Al_2_CoO_4_ electrode exhibits a significantly higher Li_2_S deposition capacity (442.2 mAh g^−1^) than Co_3_O_4_ (293.4 mAh g^−1^) and Fe_2_CoO_4_, together with a higher nucleation peak current. These observations indicate that Al_2_CoO_4_ more efficiently catalyzes the reduction of Li_2_S_4_ intermediates and accelerates Li_2_S precipitation, corroborating its faster sulfur reduction kinetics.

Overall, Al_2_CoO_4_ demonstrates a unique combination of fast redox kinetics, efficient Li_2_S nucleation, and strong polysulfide adsorption, highlighting the crucial role of modulated Co^3+^ environments in governing catalytic behavior in Li-S batteries.

**Figure 4:**
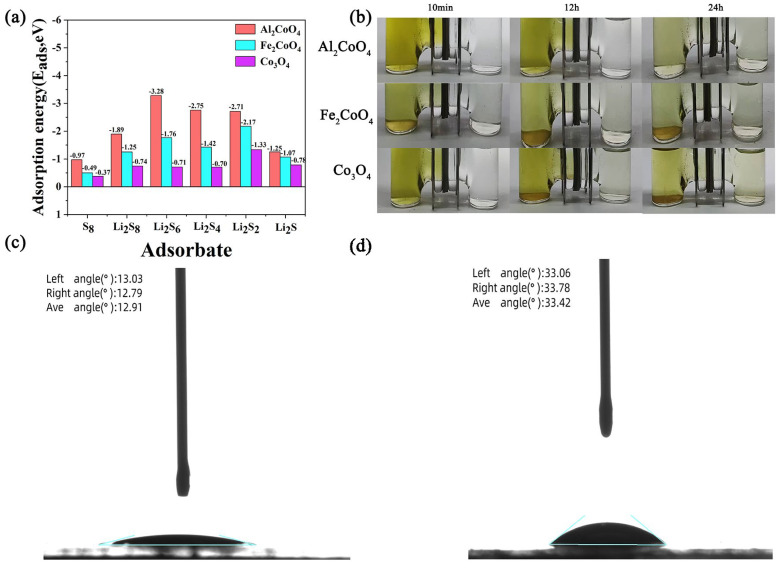
(**a**) DFT-calculated adsorption energies for LiPS and S_8_ on Co_3_O_4_, Fe_2_CoO_4_ and Al_2_CoO_4_. (**b**) Diffusion tests of Li_2_S_6_ with Co_3_O_4_@pp, Fe_2_CoO_4_ @pp and Al_2_CoO_4_@pp. Contact angle of the electrolyte drop on Al_2_CoO_4_@pp (**c**) and Commercial PP surface (**d**).

The adsorption behavior of Co_3_O_4_, Al_2_CoO_4_, and Fe_2_CoO_4_ toward lithium polysulfides (LiPS) was further evaluated through density functional theory (DFT) calculations, as shown in Figure 4a. The adsorption energies (E_ads_) of Li_2_Sₙ (n = 1, 2, 4, 6, 8) and S_8_ on Co_3_O_4_, Al_2_CoO_4_, and Fe_2_CoO_4_ were calculated using Equation (1). Al_2_CoO_4_ and Fe_2_CoO_4_ exhibit markedly larger absolute adsorption energies compared with Co_3_O_4_, confirming their stronger LiPS anchoring capability from a theoretical perspective. Specifically, for the critical soluble intermediate Li_2_S_4_, the calculated |*E_ads_*| values are 2.75 eV for Al_2_CoO_4_, 1.42 eV for Fe_2_CoO_4_, and 0.7 eV for Co_3_O_4_. This enhanced interaction arises from the formation of more stable chemical bonding between LiPS species and the exposed Co^2+^ sites, effectively suppressing polysulfide migration.

(1) $$E_{ads}=E_{adsorbate@adsorbent}-E_{adsorbate}-E_{adsorbent}$$

where $E_{ads}$ and $E_{adsorbate@adsorbent}$ are the adsorption energy (eV) and the total energy of the adsorption system (eV), respectively, $E_{adsorbate}$ is the total energy of isolated adsorbate (eV), and $E_{adsorbent}$ is the total energy of clean adsorbent (eV).

To further assess the physical blocking effect of the modified separators, Li_2_S_6_ diffusion was examined using an H-type electrolytic cell. The left chamber was filled with Li_2_S_6_ solution, while the right chamber contained LiPS-free electrolyte. The degree of polysulfide crossover is reflected by the color change in the right chamber. As shown in Figure 4b, the separators modified with Al_2_CoO_4_ and Fe_2_CoO_4_ exhibit significantly lighter solution color after 12 and 24 h compared with the Co_3_O_4_ and pristine separators, indicating markedly stronger suppression of LiPS diffusion. These results highlight the excellent polysulfide-blocking ability of Al_2_CoO_4_, providing an important mechanistic basis for designing high-performance Li–S battery separators.

The wettability of the modified separator was also evaluated by contact angle measurements. The electrolyte is a lithium–sulfur electrolyte (1 M LiTFSI in DOL/DME = 1:1 *v/v* with 2% LiNO_3_). The Al_2_CoO_4_-coated separator exhibits an average contact angle of 12.91° (Figure 4c), substantially lower than that of the commercial PP separator (33.42°, Figure 4d). The cation-engineered Al^3+^-substituted Co_3_O_4_ spinels exhibit smaller contact angles than pristine Co_3_O_4_ (12.91° for Al_2_CoO_4_ vs. 33.42° for Co_3_O_4_), indicating superior electrolyte wettability. Although no LiPS are deliberately added to the initial electrolyte, LiPS intermediates such as Li_2_S_8_ and Li_2_S_6_ are dynamically generated in the electrolyte during battery operation via the sulfur redox reaction. It has been reported that the solubility of polysulfides in the electrolyte directly governs the interfacial contact and wetting behavior between the electrolyte and the catalyst surface [39,40]. At this point, the good wettability of the initial electrolyte promotes the diffusion of generated LiPS to the catalytically active sites, reducing mass transfer resistance. It synergizes with the intrinsic adsorption-catalytic capacity of the catalyst to accelerate LiPS conversion kinetics. The significantly improved electrolyte affinity is expected to enhance interfacial wetting, reduce interfacial resistance, and facilitate faster Li^+^ transport within the cell [41].

**Figure 5:**
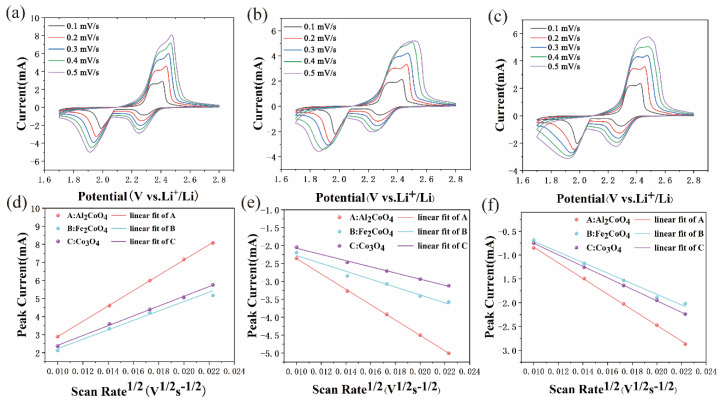
CV curves of Li–S cells with (**a**) Al_2_CoO_4_, (**b**) Fe_2_CoO_4_ and (**c**) Co_3_O_4_ modified separators at different sweep rates from 0.1 to 0.5 mV s^−1^. Linear fitting of current responses of peak 1 (**d**), peak 2 (**e**), peak 3 (**f**) and the square root of sweep rates.

The Li diffusion coefficient ($D_{{Li}^{+}}$) was calculated from cyclic voltammetry (CV) measurements (Figure 5a–c) using the Randles–Ševčík equation (Equation (2)), and the results are summarized in Table 1. The linear relationship between the peak current and the square root of the scan rate (Figure 5d–f), established by fitting, indicates that Al_2_CoO_4_ exhibits the highest $D_{{Li}^{+}}$ values at all three characteristic redox peaks, corresponding to accelerated Li^+^ diffusion, enhanced charge/mass transport, and the fastest apparent Li^+^ mass transport kinetics during the sulfur redox reaction. This result highlights Al_2_CoO_4_’s ability to facilitate overall polysulfide conversion and improve electrochemical reaction kinetics. At Peak 1, the $D_{{Li}^{+}}$ of Al_2_CoO_4_ is 2.66 times that of Fe_2_CoO_4_ and 2.45 times that of Co_3_O_4_. At Peak 2, the $D_{{Li}^{+}}$ of Al_2_CoO_4_ is 3.78 times that of Fe_2_CoO_4_ and 6.19 times that of Co_3_O_4_. At Peak 3, the $D_{{Li}^{+}}$ of Al_2_CoO_4_ is 2.18 times that of Fe_2_CoO_4_ and 1.87 times that of Co_3_O_4_. In summary, Al_2_CoO_4_ is the most favorable electrode for Li^+^ diffusion and electrochemical reactions among the three materials, providing kinetic support for its catalytic performance in lithium–sulfur batteries.

(2) $$I_{p}=(2.69\times 10^{5})n^{1.5}{D_{{Li}^{+}}}^{0.5}C_{Li}v^{0.5}$$

where $I_{p}$ is the peak current (A) and ν is the scan rate (V s^−1^), respectively, n is the number of reacting electrons (taken as 2), A represents the electrode area (cm^2^), and $C_{Li}$ is the lithium-ion concentration in the electrolyte (1 × 10^−3^ mol cm^3^).

**Figure 6:**
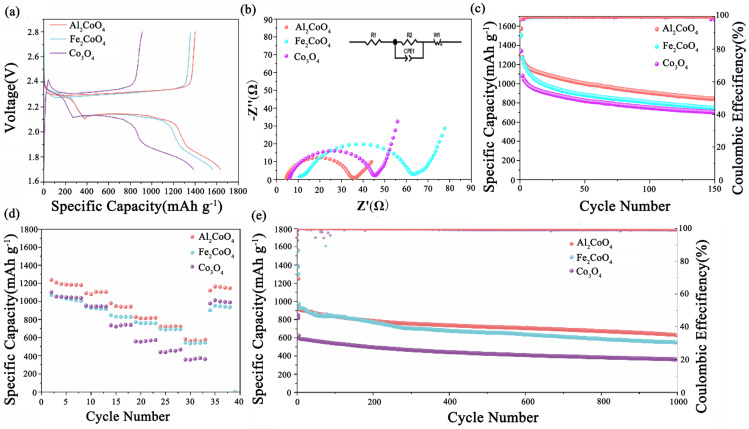
(**a**) Charge–discharge curves of batteries with different modified separators at 0.1C. (**b**) EIS of batteries with separators modified by Co_3_O_4_, Al_2_CoO_4_ and Fe_2_CoO_4_ (inset: the corresponding equivalent circuit). (**c**) Cycling capacity curves at 0.1C. (**d**) Rate performance of different cells. (**e**) Long-term cycling performance of the cells at 1C. (R_1_ is electrolyte resistance and R_2_ is the charge transfer resistance).

The charge/discharge profiles of Li–S cells with Co_3_O_4_-, Fe_2_CoO_4_-, and Al_2_CoO_4_-modified separators are presented in Figure 6a. All cells display two discharge plateaus and one charge plateau, consistent with the CV results. The Al_2_CoO_4_-modified cell exhibits the smallest polarization voltage, confirming its superior catalytic activity for LiPS conversion. Figure 6b presents the Nyquist plots and corresponding equivalent circuits of batteries with different separator modification layers. The results indicate that the R_1_ (5.37 Ω) and R_2_ (14.73 Ω) values of the Al_2_CoO_4_-based cell are significantly lower than the internal resistance of the Co_3_O_4_-based cell (R_1_ = 5.907 Ω, R_2_ = 37.35 Ω) and the Fe_2_CoO_4_-based cell (R_1_ = 11.71 Ω, R_2_ = 50.03 Ω). In addition, we conducted EIS measurements using symmetric cells. The Al_2_CoO_4_-based cell shows lower R_1_ (5.76 Ω) and R_2_ (13.45 Ω) than the Co_3_O_4_-based cell (R_1_ = 5.94 Ω, R_2_ = 38.83 Ω). These findings confirm the superior electrical conductivity and effective catalytic activity of Al_2_CoO_4_ [42,43].

The rate capability of Li–S batteries with different separator modifications was evaluated at 0.1, 0.2, 0.5, 1, 2, and 3C to investigate the effect of Al_2_CoO_4_ on LiPS conversion kinetics (Figure 6d). The cell with a Co_3_O_4_-modified separator (0.54 mg) delivered discharge capacities of 1101.9, 954.2, 734.5, 557.1, 439.8, and 356 mAh g^−1^ at increasing rates, whereas the Fe_2_CoO_4_-modified cell (0.54 mg) exhibited slightly higher capacities of 1185.9, 1023.5, 879.6, 773.9, 644.9, and 515.4 mAh g^−1^. The limited conductivity of sulfur and LiPS conversion kinetics becomes increasingly restrictive at high rates. In contrast, the Al_2_CoO_4_-modified battery (0.59 mg) achieved significantly enhanced capacities of 1238.9, 1090.6, 979.4, 826.2, 721.5, and 582.4 mAh g^−1^, indicating accelerated polysulfide redox reactions and improved lithium-ion transport. Furthermore, when the current density returned to 0.1C after high-rate cycling, the reversible capacity remained at 1120.1 mAh g^−1^, demonstrating excellent rate adaptability and stable electrochemical reversibility.

The cycling performance at low rates was also evaluated to assess practical applicability, particularly for extended discharge durations (Figure 6c). Li–S cells with Co_3_O_4_ (1.33 mg), Fe_2_CoO_4_ (1.40 mg), and Al_2_CoO_4_ (1.47 mg) modified separators exhibited initial capacities of 1080.5, 1227.5, and 1327.5 mAh g^−1^ at 0.1C, retaining 658.8, 790.3, and 883.3 mAh g^−1^ after 200 cycles, respectively. Long-term cycling at 1C further highlights the efficacy of Al_2_CoO_4_ in mitigating LiPS shuttle and enhancing redox kinetics (Figure 6e). The Al_2_CoO_4_-modified cell (1.18 mg) maintained an initial capacity of 958.6 mAh g^−1^ with a low decay rate of 0.034% per cycle over 1000 cycles, outperforming Co_3_O_4_ (622.2 mAh g^−1^, 0.042%) and Fe_2_CoO_4_ (931.1 mAh g^−1^, 0.044%). While Fe_2_CoO_4_ provides comparable initial performance, Al_2_CoO_4_ exhibits superior stability and slower capacity decay, establishing it as the optimal separator modifier for high-performance Li-S batteries.

## 4. Conclusions

In this work, three cobalt-based spinels, Co_3_O_4_, Al_2_CoO_4_, and Fe_2_CoO_4_, were successfully synthesized via a sol–gel route, where selective substitution of Co^3+^ with Al^3+^ or Fe^3+^ tailored the cationic configurations of the resulting structures. Structural analyses confirm that Al_2_CoO_4_ and Fe_2_CoO_4_ feature exclusively tetrahedral Co^2+^ sites, in contrast to Co_3_O_4_, which contains both Co^2+^ and Co^3+^. Electrochemical evaluations demonstrate that Al_2_CoO_4_ delivers the most robust catalytic activity for lithium polysulfide (LiPS) conversion, exhibiting the highest Li^+^ diffusion coefficient, the strongest LiPS adsorption capability, and the most effective suppression of the shuttle effect. As a result, Li-S batteries equipped with Al_2_CoO_4_-modified separators show an initial discharge capacity of 1327.5 mAh g^−1^ at 0.1C, retain 883.3 mAh g^−1^ after 200 cycles, and sustain 958.6 mAh g^−1^ at 1C with an ultralow capacity decay of only 0.034% per cycle over 1000 cycles. These performances surpass those of Co_3_O_4_- and Fe_2_CoO_4_-modified cells, underscoring the critical role of tetrahedral Co^2+^ sites in accelerating LiPS redox kinetics and enhancing Li^+^ transport.

## Figures and Tables

**Table 1:** Li diffusion coefficient (D_Li+_) values at different redox peak positions for different electrodes.

Electrode	Peak	Slope, *I*_*p*_/*ν*^0.5^	*D*_*Li*+_/cm^2^s^−1^
	Peak 1	0.424	6.17431 × 10^−8^
Al_2_CoO_4_	Peak 2	0.214	1.57284 × 10^−8^
	Peak 3	0.164	9.23729 × 10^−9^
	Peak 1	0.260	2.32169 × 10^−8^
Fe_2_CoO_4_	Peak 2	0.110	4.15568 × 10^−9^
	Peak 3	0.111	4.23158 × 10^−9^
	Peak 1	0.271	2.52229 × 10^−8^
Co_3_O_4_	Peak 2	0.086	2.54012 × 10^−9^
	Peak 3	0.120	4.94561 × 10^−9^

## Data Availability

The original contributions presented in this study are included in the article. Further inquiries can be directed to the corresponding authors.
